# Pointing to the Future of Language Research

**DOI:** 10.5334/joc.162

**Published:** 2021-08-23

**Authors:** Michael Long, Katherine Chia, Michael P. Kaschak

**Affiliations:** 1 Florida State University, US; 2 Department of Psychology, Florida State University, US

**Keywords:** Embodied cognition, Action and perception, Action

## Abstract

Murgiano et al. (2021) argue that indexicality and iconicity play a larger role in language than is typically acknowledged. They further call for a more situated view of language. We agree with the basic arguments laid out in their paper. In this commentary, we suggest that an embodied approach to language might provide a useful theoretical framework for developing a situated account of language.

We find much to like in Murgiano et al.’s (2021) target article. The article makes a strong case for the claim that iconicity and indexicality are more important to language use than is typically acknowledged. We agree with Murgiano et al. (2021) that adopting a situated view of language use will pay dividends for understanding child language acquisition, adult language use, and for thinking about how child language grows into adult language. Indeed, taking a situated view of language may be essential for developing a complete understanding of how language can be used to convey meaning between individuals. In this commentary, we point to one direction for developing a situated, contextual theory of language.

The idea that standard psycholinguistic theories may not easily capture the situated, contextual aspects of language has been expressed in the literature before. Clark (1983, 1997) has been skeptical that the assumptions and theories characteristic of psycholinguistic research can explain the rich and varied ways that language can be used. For example, Clark (1983) notes that many psycholinguistic models would struggle to explain the interpretation of eponymous adjectives, such as *Mike is quite Philadelphia* (where the interpretation of *Philadelphia* is uniquely anchored within a specific context). Further, Pickering and Garrod (2004) suggest that the variables that explain language behavior in controlled experiments (e.g., frequency of words or syntactic structures) might not be as relevant for explaining behavior in conversations. Others have expressed doubts about whether theories designed to explain reading times or response times in experimental settings are doing an adequate job ascertaining the interpretation that is given to the words and sentence that are processed (e.g., Ferreira & Yang, 2019). It is these sorts of issues that a situated theory of language could help to address.

What would a theory that embraces Murgiano et al.’s (2021) viewpoint look like? We believe that embodied approaches to language comprehension (e.g., Glenberg, 1997; Glenberg & Gallese, 2012) provide a good starting point. Embodied approaches see *action* as key to understanding language. This is true in at least two senses. First, language is literal action in that the speaker moves their body in order to create the speech signal and accompanying gestures. A partial understanding of language therefore constitutes interpreting the acts of the speaker – what do the speaker’s body movements, articulatory movements, prosody, and so on, suggest about what they intend to convey (e.g., Yu, McBeath, & Glenberg, in press)? Second, embodied approaches hold that linguistic meaning is grounded in our bodies’ systems of perception, action planning and emotion (e.g., Glenberg, 1997; Havas, Glenberg & Rinck, 2007). Elements of language such as words and phrases become meaningful by eliciting the retrieval of sensorimotor and emotion-based information from memory (e.g., the word “hammer” might elicit sensorimotor information about what hammers look like, how they are held and used, and so on; Barsalou, 1999; Glenberg & Gallese, 2012; Stanfield & Zwaan, 2001). Elements of language such as depictive gestures become meaningful by eliciting motor representations in the comprehender (e.g., Hostetter & Alibali, 2008). Elements of language such as deictic points become meaningful by virtue of signaling an action to be taken on the part of the comprehender (namely, *direct your eyes to where my finger is pointing*).

A virtue of embodied approaches to language is that the focus on action and sensorimotor information provides a solution to the problem of integrating the different elements of linguistic communication into a representational whole. If someone says, *“Jane threw the knife across the kitchen,”* embodied approaches propose that comprehenders use perceptual and motor information to internally simulate Jane throwing the knife, and use emotion-based information to simulate the feelings (anger) that might have caused Jane to throw the knife. If the speaker simultaneously gestures to show the nature of the throwing, the comprehender’s perception of the gestures elicits activity in their motor system that is integrated with the developing simulation of the event. Another virtue of the embodied approach is that its representational format (sensorimotor information) may provide the flexibility needed to capture the contextual, situational nature of meaning. Barsalou (1999) notes that the same sensorimotor representation (e.g., a sensorimotor representation of a hammer) can be used to derive different information in different contexts – comprehenders can focus on the weight of the hammer in a context where they need something to hold a door open, the length of the hammer in a context where they need something to do work in a tight space, or the color of the hammer in a context where they need something to decorate their office (see Masson, Bub & Warren, 2008).

An action-oriented, embodied approach to language can provide the theoretical basis from which situated language can be understood. At the same time, we acknowledge that there is quite a difference between positing that sensorimotor representations allow for flexible construal of concepts and having a fully fleshed out theory of how meaning is created in different contexts. Part of the issue may be that the embodied research program has focused quite a bit on the second sense in which language is related to action (sensorimotor representations) and largely neglected the first sense in which language is action (the act of communication itself). It may well be that understanding the range of “language actions” that a speaker might take is key to understanding how the comprehender approaches the speaker’s message. For example, when a parent picks up a long stick and says, *“Look at my sword!”* to their child, the parent’s gaze and actions can indicate to the child that *sword* is referring to the stick. The child thereby learns something about what speakers can do – they can re-name objects for the purposes of the present context. This knowledge can later help the child interpret similar contextual actions taken by speakers.

Although this proposal is far from complete, we believe that an action-oriented, embodied approach to language can provide a useful vehicle for developing Murgiano et al.’s (2021) insights into a broader theory of language learning and processing.
